# Effect of Natural Food Antioxidants against LDL and DNA Oxidative Changes

**DOI:** 10.3390/antiox7100133

**Published:** 2018-10-03

**Authors:** Sotirios Kiokias, Charalampos Proestos, Vassilki Oreopoulou

**Affiliations:** 1 REA-Research Executive Agency, B-1049 Brussels, Belgium; Sotirios.KIOKIAS@ec.europa.eu; 2 Laboratory of Food Chemistry, Department of Chemistry, National and Kapodistrian University of Athens, Panepistimiopolis Zografou, 15784 Athens, Greece; 3 Laboratory of Food Chemistry and Technology, School of Chemical Engineering, National Technical University of Athens, Iron Politechniou 9, 15780 Athens, Greece; vasor@chemeng.ntua.gr

**Keywords:** LDL-oxidation, DNA-damage, antioxidant vitamins, oxidative stress

## Abstract

Radical oxygen species formed in human tissue cells by many endogenous and exogenous pathways cause extensive oxidative damage which has been linked to various human diseases. This review paper provides an overview of lipid peroxidation and focuses on the free radicals-initiated processes of low-density lipoprotein (LDL) oxidative modification and DNA oxidative damage, which are widely associated with the initiation and development of atherosclerosis and carcinogenesis, respectively. The article subsequently provides an overview of the recent human trials or even in vitro investigations on the potential of natural antioxidant compounds (such as carotenoids; vitamins C and E) to monitor LDL and DNA oxidative changes.

## 1. Introduction to Lipid Peroxidation and Antioxidants

**Figure 1:**
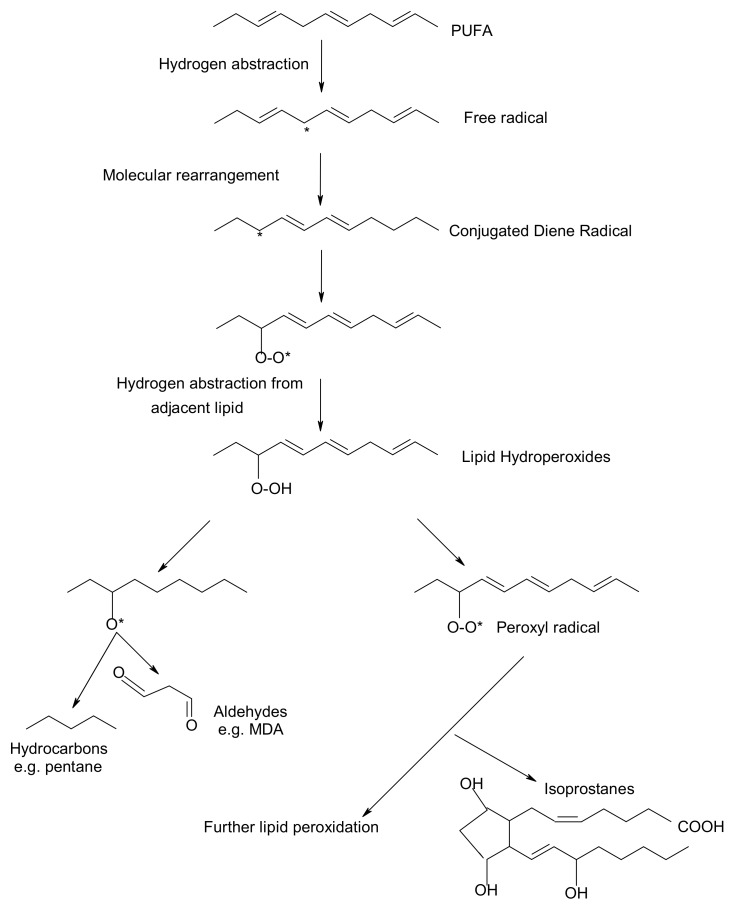
Overview of lipid peroxidation process and oxidative biomarkers (* indicates the presence of free radical).

Peroxidation of lipids, particularly of polyunsaturated fatty acids (PUFAs) is a process with marked implications: it shortens the shelf-life of food and drugs, it causes fragmentation of DNA, it damages cellular membranes and it promotes the genesis of many human diseases [1]. Lipid peroxidation is a complex biological process, initiated by free radicals, that results in the formation of conjugated dienes and lipid hydroperoxides [2]. These are usually degraded to a variety of products including alkanals, hydroxyalkenals, ketones, alkanes etc. [3]. Figure 1 reflects the lipid peroxidation process along with a few common oxidative biomarkers.

Free radicals and oxidants play a dual role as both toxic and beneficial compounds, since they can be either harmful or helpful to the body [4,5]. They are formed in tissue cells by various endogenous and exogenous pathways [6]. The ability of free radicals to structurally modify cellular components, gene expression and protein production has led to the implication of their involvement in a variety of pathological conditions, including inflammation, aging, carcinogenesis and cardiovascular diseases [7,8]. Oxidative stress exerts an adverse impact on human health. Oxygen free radicals (such as hydroxyl radicals, superoxide radicals and other active oxygen species including also singlet oxygen) adversely alter lipids, proteins, and DNA [9]. A role of lipid peroxidation and oxidative stress in the association between thyroid diseases and breast cancer has been claimed by Dominguez and Castelao (2008) [10]. Actually, overproduction of free radicals in vivo and the consequent damage to biological molecules is increasingly regarded as an important event in the development of human diseases, including arthritis, thyroid, cancer, and atherosclerosis [11].

Bhattacharyya et al. (2014) [12] noted that reactive oxygen species (ROS) are produced within the gastrointestinal (GI) tract, since ingested materials and microbial pathogens can induce oxidative injury and GI inflammatory responses involving the epithelium and immune/inflammatory cells. Therefore, further investigation on how the ROS can contribute to diverse gastrointestinal dysfunction, or manifest dual roles in cancer promotion or cancer suppression would enhance understanding of inflammation-based GI diseases and facilitate the development of new therapies [13].

Certain oxidative biomarkers have linked oxidative stress and the development of health diseases. A substantial body of evidence indicates that measurement of prostaglandine (PG)-like compounds provides a direct and reliable approach to assess oxidative damage in vivo compared with other methods such as thiobarbituric acid reacting substances (TBARS) that have been also widely studied [14]. According to Barocas et al. (2011) [15] oxidative stress measured by urine F2-isoprostane level is associated with prostate cancer. In recent years, development of immunochemical detection of 4-Hydroxynonenal (HNE)-histidine adducts opened more advanced methodological possibilities for qualitative and quantitative detection of lipid peroxidation in various human and animal tissues [16].

**Figure 2:**
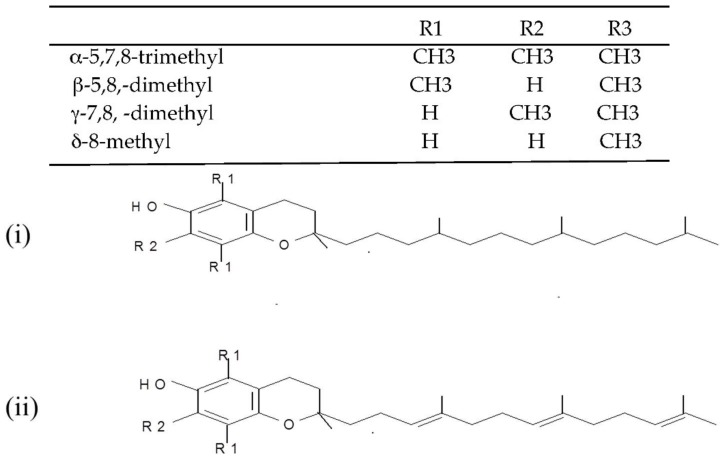
Chemical structures of the E vitamers (tocopherols-i and tocotrienols-ii).

**Figure 3:**
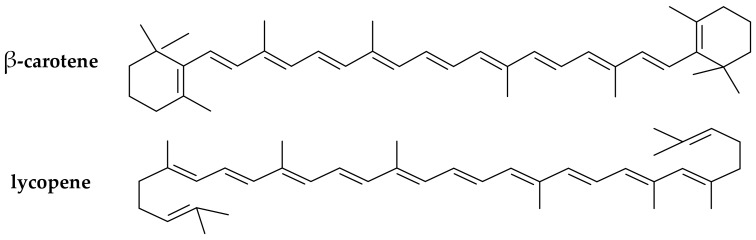
Chemical structure of the main provitamin A carotenes (β-carotene, lycopene).

To control and reduce in vivo oxidative damage, nature makes use of several types of antioxidants or radical trapping agents operating at different stages of the process [17,18]. These compounds, also known as biological antioxidants, react rapidly with free radicals and slow down the oxidative damage [19,20]. A body of evidence indicates that certain dietary compounds of plant origin can act as radical scavengers in model biological systems and in the human organism, thereby acting as dietary antioxidants [21,22]. Increased plasma total antioxidant capacity has been associated with a high consumption of fruits and vegetables rich in these vitamins, although limited information is available on whether this reflects the dietary intake of antioxidants [23]. The commonly used assays for ranking antioxidants share a common problem. Most estimates are based on methods conducted in solution and are, therefore, not necessarily relevant to processes that occur at the lipid–water interfaces in both membranes and micro emulsions, e.g., lipoproteins [24]. This review focuses on several natural compounds the levels of which in human body can be manipulated by supplements and dietary modifications. More specifically, the following ones have been reported to exert in vitro and in vivo antioxidant activities:

(i) Tocopherols and tocotrienols (vitamin E). Tocopherols and tocotrienols comprise a group of eight chromanol homologs extracted from natural sources (e.g., oils, nuts, leafy vegetables) that possess vitamin E activity in the diet [25]. They are natural monophenolic compounds with well-established antioxidant activities in food and biological systems [26]. The α-, β-, γ- and δ-tocopherols are characterized by a saturated side chain consisting of three isoprenoid units, whereas their corresponding tocotrienols have double bonds at the 3′, 7′ and 11′ position of the isoprenoid side chain [27] (structures are presented in Figure 2).

(ii) L-Ascorbic acid (vitamin C). Vitamin C, also known as ascorbic acid, occurs in all tissues of living organisms where it is responsible for the normal functioning of important metabolic processes [28]. It is very widespread in nature (e.g., in oranges, green peppers, watermelon, grapefruit) and recognized as an antioxidant nutrient with multi-functional effects depending on the conditions of the food and biological systems [29]. L-Ascorbic acid is a six-carbon weak acid with a pKa of 4.2, which is reversibly oxidized due to its enediol structure with the loss of an electron to form the free radical semihydroascorbic acid [21].

(iii) Carotenoids (provitamins A). The carotenoids are natural pigments extracted from many sources (e.g., in carrots, plums, apricots, tomatoes, spinach) that are used for various food applications. Carotenoids have been increasingly studied in the last decade for their potential to act as in vitro and in vivo antioxidants [30,31]. Dietary supplementation with certain carotenoids possessing provitamin A activity (such as β-carotene and lycopene, Figure 3) has been associated in literature with a protective role against diseases (including aging, types of cancer, cardiovascular disease, cataracts, and age-related macular degeneration) [32].

(iv) Flavonoids and phenolic acids. The current work also reports the dietary antioxidant effects of various phenolic acids available in many natural sources (e.g., in olive oil, herbs, fruits) that have been widely explored in food systems [33,34]. Flavonoids, in particular, comprise a class of phenolic compounds with well established antioxidant properties strongly related to their structure [35].

The natural occurrence and mechanisms of the antioxidant action of the above listed natural antioxidants in food-based systems have been detailed in other publications [21,36]. The present review further explores their potential effect against harmful oxidative processes (such as LDL oxidative deterioration and DNA oxidative damage), which have been widely linked in literature with the development of modern diseases including atherosclerosis and carcinogenesis.

## 2. Low-Density Lipoprotein (LDL) Oxidative Damage and Antioxidation

### Link of LDL Oxidation to Atherogenesis and Common Monitoring Methods

Human low-density lipoprotein (LDL) is defined as the population of lipoproteins, which can be isolated from plasma by ultra centrifugation within a density gradient of 1019–1063 g/L [37]. Elevated plasma concentration of LDL is a risk factor for atherosclerosis and coronary artery disease [38]. A recent body of literature has reported that atherosclerosis develops following free radical processes that cause oxidative modification of LDL [39,40]. Atherosclerosis is a progressive disease of the arterial tree that involves deposition of cholesterols in the arterial intima leading finally to a thickening of the arterial wall and reduced luminal blood flow [41]. The lipid deposited is mainly LDL, derived from the circulation. Amarowicz and Pegg [42] claimed that the exact mechanism(s) of atherosclerosis in humans remains elusive, but one theory hypothesizes that this deleterious process results from the oxidative modification of LDL.

The oxidation of LDL is a complex process during which both the proteins and the lipids undergo oxidative changes and form complex products [43]. It is a lipid peroxidation reaction driven by free radicals. Reactive oxygen species, or thiols may be released and thus participate directly in the initiation of LDL oxidation [44]. Oxidation presumably begins when a reactive radical abstracts hydrogen from a PUFA of surface phospholipids or bulk lipids in the core of the LDL particle, a reaction which in the absence of sufficient concentrations of antioxidants results in the propagation of lipid peroxidation [45].

There are various ways to measure the effect of a diet on LDL oxidation, including: the level of thiobarbituric acid reactive substances (TBARS) and lipid hydroperoxides in plasma; anion exchange chromatography and electrophoresis motility of the LDL particles; the formation of conjugated dienes (CD) at 232 nm and fluorescence spectra on the oxidation of native LDL with a chemical inducer; and the uptake of oxidised LDL by macrophages [36,46]. Given their responsiveness to targeted nutritional interventions, markers of LDL oxidation have been employed in a rapidly growing number of clinical studies for more than two decades [47].

The evaluation of LDL oxidation in vivo is difficult, and most of the investigations deal with in vitro oxidised LDL, a process accompanied by characteristic changes of physicochemical and biological properties [39,48]. The most common method for the determination of antioxidant properties of natural phenolic compounds is the LDL oxidation assay. LDL is isolated from human plasma, and oxidation is induced by Cu^2+^ ions and is monitored spectrophotometrically via the change of CD absorption at 232 nm [36,42]. Subsequently, the initiators break down the existing lipid hydroperoxides and initiate the propagation stage according to the following reactions:

(a) Cu^+^ + LOOH → Cu^2+^ +OH^−^ + LO^·^

(b) Cu^+^ + HOOH → Cu^2+^ +OH^−^ + HO

The chronology of LDL oxidation by copper ions can be divided into three consecutive time phases: lag phase, propagation phase and decomposition phase. Secondary reactions of LDL oxidation leading to aldehydes (malondialdehyde, hexanal, 4-hydroxynonenal, etc.), are accelerated by transition metal ions, such as Fe^2+^, which may catalyse the decomposition of lipid hydroperoxides to alkoxyl radicals in a Fenton-type reaction [49].

## 3. Effect of Certain Natural Antioxidants against LDL Damage/Atherosclerosis

A body of research evidence has accumulated in the last 10–15 years demonstrating how bioactive food components can protect LDL from oxidation [50].

### 3.1. Effect of Carotenoids

Earlier studies in this research field have focused on the effect of individual synthetic carotenoids, relating the good packaging of supplemented via a diet β-carotene into the lipoprotein particles, with in vivo antioxidant activities [21,51]. Kiokias and Gordon [44] were among the first researchers to explore the effects of natural carotenoid extracts against ex vivo LDL oxidation. They set up a clinical trial with 30 healthy volunteers, who were supplemented for 3 weeks with a carotenoid mixture (palm oil carotenes, lycopene, paprika, lutein, bixin in a total amount of 30 mg active carotenoid/day) and reported an increased resistance of LDL to oxidation, compared with placebo (monitored by CD at 233 nm). In a recent study, Cocate et al. [40] reported that carotenoid consumption can strongly inhibit LDL oxidative damage in healthy middle-aged men. By conducting a cross-sectional study the authors concluded that the total daily carotenoid intake (β-cryptoxanthin, lycopene, lutein plus zeaxanthin, β-carotene and α-carotene) was inversely associated (*p* < 0.05) with the plasma oxidised-LDL concentrations. Similarly, Gammone et al. [52] reported a clear effect of marine-origin carotenoids against the LDL oxidative stress.

Choi et al. [53] concluded that supplementation with astaxanthin (a polar carotenoid classified to xanthophylls) has shown positive effects, by improving the LDL oxidative stress biomarkers in a placebo-controlled study performed on overweight and obese adults. In addition, astaxanthin exerted beneficial effects to the heart, both by reducing inflammation associated with atherosclerosis and by modifying blood levels of LDL-cholesterol and high-density lipoprotein (HDL)-cholesterol [54]. Ciccone et al. [55] noted that, despite some contradictions, there are many clinical and epidemiological data supporting the anti-inflammatory action and protective effect of carotenoids against cardiovascular events, with findings being more favourable for the natural carotenoids than for the synthetic ones.

Furthermore, a number of clinical trials concluded that a short term dietary supplementation of healthy volunteers with lycopene-rich products (providing ~30–50 mg lycopene/day) increases the resistance of LDL to oxidative deterioration [56,57]. On the contrary, other researchers have supported that relatively high doses of carotenoid supplements (e.g., 60–150 mg of active carotenoids/day) may result in excessively enriched LDL particles with carotenoid metabolites, thereby leading to even an increased susceptibility of LDL to oxidation rather than to any protective effect [21,36].

Overall, by analysing the existing evidence, it can be hypothesized that relatively low levels of carotenoid enrichment by dietary supplementation may be more effective at inhibiting oxidation of LDL ex vivo than larger in vitro enrichments that fails to produce any beneficial effect.

### 3.2. Effect of Vitamin C

Although earlier studies in smoking subjects, did not report any significant effect of dietary supplementation with vitamin C on LDL oxidation, more recent research results have shown a protective activity of ascorbic acid against LDL oxidation [58,59]. Hillstrom et al. [60] demonstrated that vitamin C inhibits lipid oxidation in HDL and preserves the antioxidant activity associated with this lipoprotein fraction. Shariat et al. [61] evaluated the in vitro antioxidant effects of various vitamins on LDL oxidation and concluded that vitamin C (50–200 mM) is able to inhibit LDL oxidation mediated by myeloperoxidase with a concentration dependent effect.

### 3.3. Effect of Vitamin E

Jacobson et al. [62] supplemented hyperlipedemic rabbits with 500 mg α-tocopherol/kg for 24 weeks, reporting an increased resistance to LDL oxidation (lag time of LDL oxidation in the treated group almost 2 times higher than in the placebo). Parameswari et al. [63] reported a beneficial effect of vitamin E, on the copper ion-induced oxidation of LDL, isolated from the serum of chronic renal failure and renal transplanted patients. Ghaffari and Ghiasvand [48] studied the effect of different concentrations of α-tocopherol on in vitro cupric ions induced oxidation of LDL. Their results revealed that α-tocopherol (0–100 μmol/L) may decrease free radicals in LDL and, thus, the rate of LDL oxidation by cupric ions. However other researchers did not observe any beneficial effects of tocopherols supplementation. Car et al. 2018 [64] supported that α-tocopherol can even act as pro-oxidant to facilitate lipid peroxidation in LDL, an adverse effect that can be prevented when ascorbate is acting as a coantioxidant. Dotan et al. 2009 [65] has further challenged the beneficial effects of tocopherols by claiming that indiscriminate, high doses of vitamin E supplementation results in increased mortality and should not be recommended to the general public.

Niki [66] supported that vitamin E and other antioxidants inhibit LDL oxidation efficiently in vitro; however, human clinical trials with vitamin E have not yielded positive results. An explanation for that could be that LDL oxidation proceeds by multiple pathways mediated not only by free radicals but also by other non-radical oxidants and vitamin E is effective only against free radical mediated oxidation. Furthermore, Niki (2011) [67] has provided an additional explanation for the non-protective effect of vitamin E against LDL deterioration in human clinical trials claiming that in contrast to animal experiments, vitamin E is given at the latter stage where oxidation is no more important. Free radicals must play a crucial role in the pathogenesis of atherosclerosis and vitamin E should be effective if given at right time to right subjects.

### 3.4. Effect of Flavonoids, Phenolic Acids and Antioxidant Mixtures

The functional groups of flavonoids attached to the three-ring system has been reported to trigger a positive impact against LDL oxidation [21]. In a study by Naderi et al. [68] the susceptibility of LDL to in vitro oxidation was monitored by the change in 234-absorbance in the presence and absence of several pure flavonoids at different concentrations. According to the results, flavonoids significantly protected against in vitro LDL oxidation, with genistein, morin and naringin exerting a stronger inhibitory activity than quercetin or apigenin.

Amarowicz and Pegg [42] reported that studies on LDL oxidation (monitored by measurement of the generation of conjugated dienes and trienes) confirmed (i) the antioxidant properties of several extracts obtained from plant materials (e.g., grapes, berries, orange, grapefruit, coffee, tea, chocolate, olives, nuts); and (ii) the in vitro protective effect of phenolic compounds (e.g., luteolinidin, apigenidin, caffeic acid, chlorogenic acid, catechin, quercetin, rutin) against LDL oxidation. Carmeli and Fogelman [69] conducted a study to determine the effect of a natural polyphenolic isoflavone (glabridin) on LDL oxidation, by measuring the formation of TBARS, and observed that after oral administration of a glabridrin-rich extract of licorice-root to healthy subjects for 6 months, their oxidative stress level as well as plasma LDL oxidation reduced by 20%. Lam et al. [70] examined the antioxidant effects of selected phenolic compounds from natural sources. According to their results, 6-gingerol and rhapontin were found to exhibit strong inhibition against in vitro lipid peroxidation in LDL induced by 2,2-azobis(2-amidinopropane) dihydrochloride (AAPH), while barbaloin possessed weaker effects.

Costa-Mugica et al. [71] reported that lyophilized aqueous extracts and phenolic-rich fractions of seaweed (H. Incrassata) significantly inhibited LDL oxidation when evaluated by using heparin-precipitated LDL exposed to Cu^2+^ ions with AAPH as the free radical generator. The authors claimed that the observed effect could be related to the antioxidant potential of the polar phenolic fractions. In addition, Singh et al. [72] reported that LDL oxidative modification was significantly higher (*p* > 0.001) in diabetic patients as compared to control subjects. An explanation could be provided by the finding that the plasma antioxidant capacity had been decreased significantly (*p* > 0.001) in the unregulated diabetic group compared to the control group.

Aviram et al. [73] reported that dietary supplementation of polyphenol-rich pomegranate juice to atherosclerotic mice significantly inhibited the development of atherosclerotic lesions and this may be attributed to the protection of LDL against oxidation. On the contrary, Carru et al. [74] have recently reported that an extract of roasted coffee (rich in various phenolic compounds) acted as pro-oxidant accelerating the in vitro LDL oxidation triggered by copper sulphate.

Chu and Liu [75] developed a model based on peroxyl radical-initiated LDL oxidation, by use of the water-soluble free radical initiator AAPH, to assess the free radical scavenging capacity of antioxidants and extracts of natural products. The authors reported that all the tested concentrations of vitamin C and E and apple extract resulted in partial suppression and delay of LDL oxidation in terms of headspace hexanal, as a major decomposition product measured by a headspace gas chromatograph.

A summary of the most recent clinical trials exploring on the antioxidant effect of vitamins and natural antioxidants against LDL oxidative modification is given in Table 1.

## 4. DNA Oxidative Damage and Antioxidation

### 4.1. Link of DNA Oxidative Damage to Carcinogenesis and Common Monitoring Methods

Cancer is a leading cause of disease burden throughout the world. Many cancers develop as a result of exposure to both lifestyle and environmental factors that are potentially modifiable, with oxidative stress playing an important role in their pathogenesis [76]. The complex series of cellular and molecular changes participating in cancer development are mediated by a diversity of endogenous and exogenous stimuli [77]. A relatively new but promising strategy for cancer prevention involves the use of natural dietary compounds and shows promising results in vitro and in vivo, i.e., in animal and human clinical trials [17,78]. In regard to cancer, probably the most important target for reactive oxygen species (ROS) is DNA. DNA is a molecule prone to damage from exogenous and endogenous sources with important consequences for mutagenic and carcinogenic processes [79]. Oxidative DNA damage caused by ROS makes a significant contribution to genomic instability, carcinogenesis and cellular ageing, thereby provides a valuable biomarker of overall oxidative stress [80]. At the molecular level, damage to DNA can take many forms, ranging from specifically oxidised purine and pyrimidine bases (more than 20 such oxidative lesions have been identified), to gross DNA changes such as strand breaks, sister chromatid exchange, and the formation of micronuclei [81]. Oxygen radicals may attack DNA at either sugars or bases, giving rise to a large number of damaged products. According to one of the proposed oxidative mechanisms, hydrogen peroxide can cause DNA strand breakage, by generation of the hydroxyl radical (OH·) close to the DNA molecule, via the Fenton reaction [36]:

H_2_O_2_ + Fe^2+^ → OH^−^ + OH^−^ + Fe^3+^

This may result in DNA instability, mutagenesis and ultimately carcinogenesis. Specific DNA oxidation products accumulate depending on the ROS involved, its rate of production, and the cell’s ability to protect or repair its DNA insult [82,83]. According to researchers in this field [84,85], the C-8 hydroxylation of guanine is one of the most frequent DNA base modifications, usually generated when DNA is directly oxidised by hydroxyl radicals or peroxynitrite.

Various analytical techniques exist for the measurement of oxidative damage to DNA including gas chromatography (GC) or liquid chromatography (LC) with mass spectrometry (MS) that simultaneously measure numerous products, and provide positive identification and accurate quantification [7,21]. 8-hydroxy-2’-deoxyguanosine (8-OH-dG), its corresponding base 8-oxo-guanine (oxoG) and 8-oxo-adenine have been used as useful markers of oxidative DNA damage [77]. There are two main reasons, however, for the particular popularity of 8-OH-dG as oxidative biomarker [86]: (a) it can be easily detected using high-performance LC (HPLC) with an electrochemical detector, and (b) 8-OH-dG, itself has biological significance for DNA basis tranvsersions at DNA replication. However, there have been doubts regarding the accuracy of the 8-OH-dG amounts detected because of the possibility of artifact production as well as sample oxidation during the preparation processes.

The Japan Institute for the Control of Aging [87] has developed an in vitro enzyme-linked immunosorbent assay (ELISA) for quantitative measurement of the oxidative DNA adduct 8-OH-dG in tissue or urine samples. This technique makes use of a specific monoclonal antibody that recognises both the modified base and deoxyribose structure of 8-OH-dG, whereas it does not cross react with the original four deoxyribonucleosides, other DNA base modified products (e.g., oxo-adenine), or urine components (uric acid, creatine, etc.) [79]. The basic advantages of this analytical tool include easy operation, high sensitivity, speed, and large sample capacity. As creatinine levels in urine are a measure of the concentration of the fluid, they co-vary with 8-OH-dG and determination of urinary creatinine is important in expressing the level of this DNA oxidative product with this method.

An alternative approach to the determination of oxidised DNA bases makes use of repair enzymes (e.g., endonuclease III) to introduce strand breaks at sites where oxidised bases are present and estimation by electophoretic techniques [88]. This method is a **single** cell gel electrophoresis assay-SCGE, (most widely known as COMET assay). It is a very sensitive and valuable technique that allows the detection of intercellular differences in DNA by measuring the single/double-strand DNA breaks [89]. The sensitivity and specificity of the COMET assay are greatly enhanced if the nucleoids are incubated with bacterial repair endonucleases that recognize specific kinds of damage in the DNA and convert lesions to DNA breaks, increasing the amount of DNA in the comet tail [90]. This in vitro DNA repair assay has been modified for use in animal tissues, specifically to study the effects of aging and nutritional intervention on repair [91]. Previous studies on healthy individuals have shown that a high intake of fruits and vegetables can decrease oxidative DNA damage as measured by the alkaline comet assay [92]. The topic will be further explored in the next section.

### 4.2. Effects of Natural Antioxidants against DNA Damage/Carcinogenesis

#### 4.2.1. Effect of Carotenoids

Astley et al. [93] conducted three independent clinical trials where healthy male volunteers supplemented their habitual diets with lutein, beta-carotene or lycopene (natural isolate capsules, 15 mg/d, 4 weeks). Their results suggest that the carotenoids are capable of exerting two overlapping but distinct effects: antioxidant protection by scavenging DNA-damaging free radicals and modulation of DNA repair mechanisms.

Herrero-Barbudo et al. [94] examined the effect of dietary intervention of 10 humans with lutein-enriched fermented milk (containing lutein and lutein esters at concentration 4–8 mg free lutein/100 mL) on DNA-induced damage. By using the COMET assay they concluded that the regular consumption of lutein-enriched fermented milk resulted in a significant increase in serum lutein levels and this change was associated with an improvement in the resistance of DNA to endogenous damage and the capacity of DNA repair in lymphocytes. Furthermore, a significantly inverse correlation between plasma lutein (increase) and change in lymphocyte 8-OH-dG was reported by Haegele et al. [95] following 2 weeks dietary intervention of 37 healthy women with 12 servings of fruits and vegetables per day. Cocate et al. [40] conducted a cross-sectional study with the participation of 296 apparently healthy middle-aged men to assess the potential relationships of carotenoid intake with lipid and oxidative stress markers. In conclusion, the total daily carotenoid intake based on five investigated carotenoid types (β-cryptoxanthin, lycopene, lutein plus zeaxanthin, β-carotene and α-carotene) was inversely associated with the production of urinary 8-OH-dG as oxidative stress biomarke (*p* < 0.05).

Kiokias and Gordon [44] conducted a double-blind, placebo-controlled cross over study with 30 healthy subjects. Following dietary supplementation of 30 mg carotenoid mixture/day (α-, β-carotene, lycopene, paprika, lutein, bixin, total amount) they reported a significant effect against production of urinary 8-OH-dG estimated by the use of ELISA test. Furthermore, Barcelos et al. [96] following a carotenoid dieterary intervention in rats, reported that bixin and norbixin protect against DNA-damage and alterations of redox status, induced by methylmercury exposure in vivo. They claimed that dietary consumption of these specific carotenoids may protect humans against the DNA damage caused by exposure to organic mercury.

#### 4.2.2. Effect of Vitamin C

In an earlier study, Noroozi et al. [97] reported that pre-treatment of human lymphocytes with vitamin C produced dose-dependent reductions of oxidative DNA damage. Subsequently, Kadirvel et al. [98] reported that supplementation with ascorbic acid significantly prevents the arsenic-induced protein oxidation and DNA damage in rats. More recently, Kontek et al. [99] reported that vitamin C (in a concentration range 10–100 μm) caused a clear protecting effect against DNA damaging. More specifically, they concluded that vitamin C modulates DNA damage induced by hydrogen peroxide in human colorectal adenocarcinoma cell lines (HT29), estimated by COMET assay in vitro (decrease ~30%). Asgard [100] reported that high plasma levels of ascorbate reduced the levels of oxidative DNA damage (8-oxodG) in mononuclear white blood cells.

#### 4.2.3. Effect of Vitamin E

Steady state estimates of cellular DNA oxidation, in general have provided support for an antioxidant role of vitamin E [101]. Makpol et al. [102] observed that α-tocopherol protected against hydrogen peroxide-induced DNA damage and this protection was affected by the donor’s age. Fantappiè et al. [103] assessed susceptibility to lipid peroxidation and oxidative DNA damage in the human hepatocellular carcinoma, by measuring the concentration of TBARS and 8-OH-dG at basal and after experimental conditions. They reported that vitamin E protects DNA from oxidative damage in human hepatocellular carcinoma cell lines. Kadirvel et al. [98] examined the effects of α-tocopherol against the DNA damage induced by arsenic in rats and reported that it can significantly improve the molecular alterations. Ragin et al. [104] conducted a human trial showing that an intake of food rich in α-tocopherol could decrease levels of DNA oxidative adducts. Asgard [100] reported a significant decrease of catechol-induced (1 mM) general DNA damage in the presence of 20 μM of α-tocopherol. By contrast, De Oliveira et al. [105] supported that dietary supplementation with α-tocopherol can even induce DNA oxidative stress.

#### 4.2.4. Effect of Flavonoids and Phenolic Acids

A body of research evidence links consumption of phenolic compounds with inhibition of DNA oxidative changes [106,107]. In a very recent study, Vazhappilly and Vasantha [108] investigated the efficacy of an apple flavonoid fraction against the DNA damage in normal human bronchial epithelial cells. The results revealed an increased level of DNA damage proteins in carcinogen-treated cells that was significantly (*p* ≤ 0.05) inhibited in the flavonoid-pretreated cells. Rusac et al. [109] investigated into flavonoid-DNA interactions and cytotoxic potential of flavonoids in human peripheral blood lymphocytes. Luteolin, followed by apigenin and kaempferol, was shown to be the most effective in protecting DNA from oxidative damage induced by hydrogen peroxide. However, the examined flavonoids also induced DNA damage, indicating their prooxidative capacity. The authors claimed that the balance between the antioxidant and prooxidant character was strongly dependent on flavonoid concentration and the incubation period. By contrast, Tsai et al. [110] reported that propolis flavonoids (such as galangin, chrysin, and pinocembrin) did not protect but instead accelerated hydrogen peroxide-induced DNA damage. They noted, though, that propolis induces oxidative DNA damage that is subject to repair, and propolis-treated cells can even show a lower level of DNA damage when challenged with another oxidative agent such as amoxicillin. Sevgi et al. [111] reported that certain phenolic acids (ferulic, caffeic, rosmarinic, and vanillic acids) protected plasmid DNA from oxidative damage in the presence of hydrogen peroxide and UV. Similarly, Fabiani et al. [112] observed that oxidative DNA damage in human blood mononuclear cells and HL60 cells was prevented by extracts of olive oil, hydroxytyrosol, and other olive phenolics. Lodovici et al. [113] reported that a few natural phenolic acids (e.g., 2-coumaric, 3-coumaric acids), commonly present in food, exert interesting protective activity against DNA oxidation in vitro and deserve further consideration as effective antioxidants in vivo. Other researchers [114,115] confirmed in vitro effects of various flavonoids (e.g., quercetin, myricetin, luteolin, morin and cyanidin) against DNA oxidative damage in terms of oxidative adducts including 8-OH-Dg. Barcelos et al. [116] observed that dietary supplementation of rats with quercetin (0.5–50 mg/kg/bw/day), over 45 days, resulted in an increased protection against DNA damage induced by methyl mercury. In a very recent study, Srivastava et al. [117] reported that quercetin led to ~5 fold increase in the life span in tumor bearing mice compared to that of untreated controls. The authors claim that quercetin interacts with DNA directly and could be one of the mechanisms for inducing apoptosis in both cancer cell lines and tumor tissues by activating the intrinsic pathway. These data suggests that quercetin can be further explored for its potential to be used in cancer therapeutics and combination therapy.

#### 4.2.5. Effect of Antioxidant Mixtures

By contrast with the data presented in the previous section about inhibition of LDL oxidation, antioxidant combinations have not been consistently reported to protect against DNA oxidative damage. Asgard [100] reported that supplementation of 47 type-2 diabetes subjects for 12 weeks with 16 capsules/day (mixture of β-carotene and α-tocopherol) did not exert any inhibitory effect against DNA oxidative stress. Similarly Rytter et al. [118] concluded that biomarkers of oxidative stress in overweight men are not influenced by a dietary supplementation with antioxidant combinations. An overview of the most recent clinical studies exploring on the antioxidant effect of natural compounds against DNA oxidative damage is given in Table 2.

## 5. Main Conclusions/Future Work

(i) A review of the studies in this research field revealed strong antioxidant effects of various dietary compounds extracted from natural sources (e.g., ascorbic acid, tocopherols, carotenoids) against LDL oxidative modification and DNA oxidative damage both in vitro or in vivo. Being lipophilic in nature, most of these dietary antioxidants are located within lipoproteins and in cellular membranes, and therefore present an efficient concentration to protect against particular oxidative stress conditions.

(ii) A shift from an antioxidant to a prooxidant character of the examined compounds may in certain cases occur due to experimental conditions (e.g., concentration of tested compounds) or structure-related causes. For instance, in environments with a high load of oxidative stress, certain antioxidants (e.g., carotenoids) may exert a prooxidative effect increasing the level of oxidative DNA damage in the cell system. In particular for β-carotene, authors have concluded that an explanation for the lack of antioxidant activity in high concentrations could be due to the fact that this compound can readily undergo autoxidation, thereby generating reactive oxygen species capable of initiating further harmful oxidation processes.

(iii) A few studies have focused on the effect of natural antioxidant mixtures rather than on individual synthetic compounds. Certain antioxidant combinations have been reported to protect efficiently against LDL oxidative modification. Such an enhanced beneficial effect of antioxidant mixtures may be partly related to biological interactions between compounds with various modes of action and possible synergistic effects, in particular between compounds with different structures and modes of action (e.g., terpenoid carotenoids vs phenolics). As evidenced by the existing literature, the antioxidant synergies seem to work more efficiently against LDL than DNA oxidative changes.

(iv) Further work is required in this field to explore the effects of dietary natural antioxidants and, more specifically, how their activity is influenced by (i) their concentrations that may induce an antioxidant/prooxidant character; and (ii) their actual location of the antioxidants in the cell (such as in the cell membranes and cytoplasma or closer to the DNA).

(v) In addition to the in vitro investigations, there is a particular need to conduct more clinical dietary trials in humans that would (i) further elucidate the effect of natural antioxidants (and in particular of their mixtures) against oxidative stress; and (ii) set the optimal conditions for these dietary compounds to exert a protective effect that could potentially lead to new therapeutic strategies against certain types of cancers and cardiovascular diseases.

## Figures and Tables

**Table 1:** Selection of studies on the effect of natural antioxidants against low-density lipoprotein (LDL) oxidative damage (conducted during the last 15 years).

Researchers/Study (*per Chronological Order*)	Experimental Conditions	Effect on LDL Oxidation
Kiokias and Gordon (2003) [44]/Clinical trial	Supplementation of 30 healthy volunteers with 30 mg active carotenoid/day (mixture of palm oil carotenes, lycopene, paprika, lutein, bixin) for 3 weeks.	Reduced ex vivo copper-induced LDL oxidation monitored by measurement of conjugated dienes at 233 nm.
Naderi et al. (2003) [68]/In vitro study	Model of LDL oxidation monitored by the change in 234-absorbance in presence of various flavonoids.	Genistein, morin and naringin exterted a stronger inhibitory activity than quercetin or apigenin.
Jacobson et al. (2004) [62]/Clinical trial	Supplementation of hyperlipedemic rabbits with 500 mg α-tocopherol/kg for 24 weeks.	Increased resistance to LDL oxidation observed in carotenoid treated rabbits (lag time of LDL oxidation in treatment group almost 2 times higher than in the placebo).
Lam et al. (2009) [69]/In vitro study	Model of lipid peroxidation in LDL induced by AAPH radical initiator.	Selected phenolic compounds from dietary sources (6-gingerol and rhapontin) were found to exhibit a strong inhibitory effect against LDL oxidation whereas barbaloin possessed weaker effects.
Carmeli and Fogelman (2009) [69]/Clinical trial	Supplementation of 10 healthy subjects for 6 months with a licorice-root extract rich in the isoflavone glabridin.	LDL oxidative stress was reduced by 20% in terms of TBARS in the treatment group compared to baseline.
Ghaffari and Ghiasvand (2010) [48]/In vitro study	Model of LDL oxidation induced by cupric ions.	α-tocopherol (in the range 0–100 μmol/L) reduced LDL oxidative deterioration.
Choi et al. (2011) [53]/Clinical trial	Supplementation of 27 overweight and obese adults with the carotenoid astaxanthin in a placebo-controlled study performed for 12 weeks.	The treatment group presented lower levels of LDL oxidative biomarkers compared to the placebo group.
Costa-Mugica et al. (2012) [71]/In vitro study	Model of heparin-precipitated LDL exposed to Cu^2+^ ions with AAPH as the free radical generator.	Lyophilized aqueous extracts and phenolic-rich fractions of seaweed (*H. incrassata)* significantly inhibited LDL oxidation.
Shariat et al. (2013) [61]/In vitro study	Model of LDL oxidation mediated by myeloperoxidase	Vitamin C inhibited LDL oxidation with a concentration dependent effect (50–200 mM).
Cocate et al. (2015) [40]/Clinical trial	Supplementation of 296 healthy middle-aged supplemented with a carotenoid mixture (β-cryptoxanthin, lycopene, lutein plus zeaxanthin, β-carotene and α-carotene).	The daily carotenoid intake was inversely associated (*p* < 0.05) with the plasma oxidised-LDL concentrations.

**Table 2:** Selection of studies on the effect of natural antioxidants against DNA oxidative damage (conducted during the last 15 years).

Researchers/Study (per chronological order)	Experimental Conditions	Effect on LDL Oxidation
Kiokias and Gordon (2003) [44]/Clinical trial	Supplementation of 30 healthy volunteers with 30 mg active carotenoid/day (mixture of α,β-carotene, lycopene, paprika, lutein, bixin) for 3 weeks.	Carotenoids caused a significant reduction (*p* < 0.05) of in vivo DNA oxidative damage in terms of 8-OH-dG as biomarker.
Astley et al. (2004) [93]/Clinical trial	Supplementation of healthy males with 15 mg/d lutein, β-carotene or lycopene (natural isolate capsules) for 4 weeks (3 independent clinical trials).	Carotenoids presented an antioxidant Character protection by scavenging DNA-damaging free radicals modulation of DNA repair.
Fantappiè et al. (2004) [103] /In vitro study	Model of oxidative DNA damage in the human hepatocellular carcinoma.	Vitamin E protected DNA from oxidative damage as evidenced by the concentration of TBARS and 8-OH-dG biomarkers after carotenoid treatment.
Fabiani et al. (2008) [112]/In vitro study	Model of oxidative DNA damage in human blood mononuclear cells and HL60 cells.	Extracts of olive oil, hydroxytyrosol, and other olive phenolic compounds exerted a strong inhibitory effect against DNA damage.
Rusac et al. (2010) [109]/In vitro study	Model of flavonoid-DNA interactions in human peripheral blood lymphocytes.	Certain flavonoids (luteolin, apigenin and kaempferol) were shown effective in protecting DNA from oxidative damage induced by hydrogen peroxide.
Barcelos et al. (2012) [96]/Clinical trial	Rats were treated orally with quercetin (0.5–50 mg/kg/bw/day), over 45 days.	Quercetin concentrations (5.0 and 50.0 mg/kg/bw/day) were found to protect against DNA damage.
Herrero-Barbudo et al. (2013) [94] /Clinical trial	Supplementation of 10 humans with lutein-enriched fermented milk (containing lutein and lutein esters at concentration 4–8 mg free lutein/100 mL).	A significant increase in serum lutein levels, was associated with an improved resistance to DNA damage.
Cocate et al. (2014) [40]/Clinical trial	Supplementation of 296 healthy middle-aged supplemented with a with carotenoid mixture (β-cryptoxanthin, lycopene, lutein plus zeaxanthin, β-carotene and α-carotene).	The daily carotenoid intake was inversely associated with the production of urinary 8-OH-dG as oxidative stress biomarker (*p* < 0.05).
Asgard (2014) [100]/Clinical trial	47 type-2 diabetes subjects supplemented for 12 weeks with 16 capsules/day (mixture of β-carotene + α-tocopherol).	Dietary supplementation did not affect the levels of biomarkers of oxidative stress and inflammation, despite substantially increased plasma concentrations of antioxidants.
Sevgi et al. (2015) [111]/In vitro study	Model of plasmid DNA oxidative damage in the presence of hydrogen peroxide and ultraviolet (UV) light.	Tested phenolic acids (ferulic, caffeic, rosmarinic, and vanillic acids) inhibited DNA damage.
